# Haematology and biochemistry of the San Cristóbal Lava Lizard (*Microlophus bivittatus*)

**DOI:** 10.1093/conphys/coy046

**Published:** 2018-08-17

**Authors:** Randall Arguedas, David Steinberg, Gregory A Lewbart, Diane Deresienski, Kenneth J Lohmann, Juan Pablo Muñoz-Pérez, Carlos A Valle

**Affiliations:** 1 Zoológico Nacional Simón Bolívar, Barrio Amón, Calle 13, Apdo. 11594-1000, San José, Costa Rica; 2 Department of Biology, University of North Carolina, Chapel Hill, NC 27599, USA; 3 College of Veterinary Medicine, Department of Clinical Sciences, North Carolina State University, 1060 William Moore Drive Raleigh, NC 27607, USA; 4 Galápagos Science Center GSC (Universidad San Francisco de Quito-University of North Carolina at Chapel Hill), Av. Alsacio Northia, Isla San Cristobal, Galápagos, Ecuador; 5 Universidad San Francisco de Quito USFQ, Galapagos Science Center GSC, Colegio de Ciencias Biológicas y Ambientales COCIBA, Campus Cumbayá Av. Diego de Robles S/N e Interoceánica, Galápagos Casilla Postal 17-1200-841, Quito 170901, Ecuador

**Keywords:** Biochemistry, Galápagos, haematology, health assessment, *Microlophus bivittatus*, San Cristóbal lava lizard

## Abstract

The San Cristóbal lava lizard, *Microlophus bivittatus*, is one of nine species of lava lizards endemic to the Galápagos Islands of Ecuador. No information presently exists about baseline health parameters for any of these species. We analysed blood samples drawn from 47 lizards (25 males and 22 females) captured at two locations on San Cristóbal Island. A portable blood analyser (iSTAT) was used to obtain near-immediate field results for total CO_2_, lactate, sodium, potassium, ionized calcium, glucose and haemoglobin. Standard laboratory haematology techniques were employed for differential white blood cell counts and haematocrit determination. Body temperature, heart rate and body measurements were also recorded. We found significant differences in haematocrit values between males and females. The values reported in this study provide baseline data that may be useful in detecting changes in health status among lava lizards affected by natural disturbances or anthropogenic threats. Our findings might also be helpful in future efforts to demonstrate associations between specific biochemical or haematological parameters and disease. Because there are several related species on different islands in the Galápagos archipelago, comparisons between populations and species will be of interest. Lay Summary:Haematology and biochemistry values of the San Cristóbal lava lizard *Microlophus bivittatus*, along with several other health parameters (morphometrics and temperature), are reported for the first time.

## Introduction

The measurement of biochemical and haematological parameters can serve as a valuable tool for evaluating and monitoring the health of wild reptile populations (Stacy *et al.*, 2011; Campbell, 2014). However, a major obstacle to conducting wildlife health assessments is a lack of baseline data against which new data can be compared (Calle *et al.*, 2017). Without an understanding of typical species-specific (or taxon-specific) variation in biochemical and haematological parameters, researchers are unable to identify potential effects of disease, injury, pollutants or other changing environmental conditions (Lewbart *et al.*, 2015). This issue is particularly important for regions with large numbers of endemic species that are experiencing rapid change, such as the Galapagos Archipelago.

**Figure 1:**
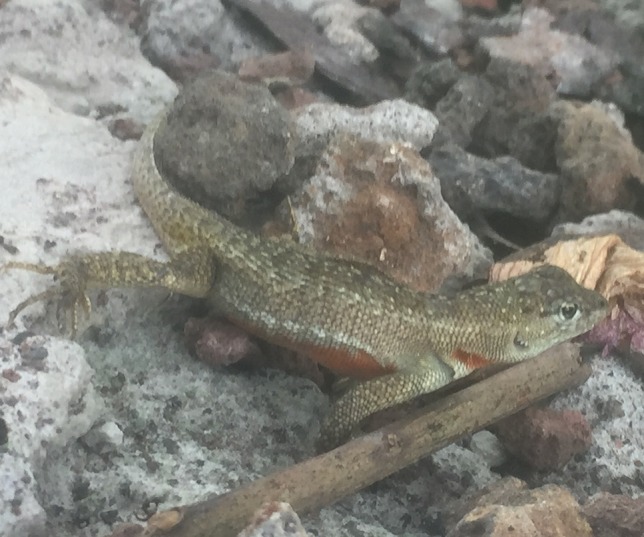
Female individual of a San Cristóbal Lava Lizard (*Microlophus bivittatus*).

The Galapagos Archipelago has nine endemic species of lava lizard in the South American genus *Microlophus* (Family: Tropiduridae) (Benavides *et al.*, 2009; Jiménez-Uzcátegui *et al.*, 2017). *Microlophus bivittatus* (Fig. 1), which is restricted to the island of San Cristóbal and nearby Islote Lobos, inhabits xeric, low-elevation areas, such as coastal scrubland and rocky beaches. This lizard is listed as near threatened (Márquez and Cisneros-Heredia, 2016) and is particularly vulnerable to predation by feral cats (Carrión Avilés, 2012; Carrion and Valle, 2018). At present, no blood chemistry or haematology data have been acquired for the genus *Microlophus*, which complicates efforts to evaluate or monitor the health of wild lava lizard populations. In this study, we report baseline biochemical and haematological values, along with basic physiological measures, for the San Cristóbal lava lizard.

## Methods

### Ethics statement

This study was conducted on San Cristóbal Island in the Galápagos archipelago of Ecuador as part of a population health assessment authorized by the Galápagos National Park Service (Permit No. PC-23-17, PI Carlos A. Valle, PhD) and approved by the Universidad San Francisco de Quito ethics and animal handling protocol. All handling and sampling procedures were consistent with standard vertebrate protocols and veterinary practices.

### Capture and marking

Lizards were captured in locations within 2 km of the Galapagos Science Center on San Cristobal Island. Twenty-six lizards (*N*_male_ = 13, *N*_female_ = 13) were captured along a walking trail to Punta Carola (0°53′32″ and 89°36′43″) on 5 July 2017. On the following day, 21 lizards (*N*_male_ = 12, *N*_female_ = 9) were captured along Tijeretas trail (0°53′34″and 89°36′32″).

Each lizard was captured by noose, placed in a cloth bag, and transported to a temporary field laboratory for processing (see below). Afterwards, to avoid recapturing the same individuals, each was marked on its dorsum with a spot of non-toxic, red acrylic paint used previously in other studies (Berriozabal *et al.*, 2017; Keogh *et al.*, 2012; Pasek and Collver, 2001) and known to wash or rub off within 10 days of application (C.A. Valle, personal observation). Because the study was conducted outside of the breeding season and the paint fades quickly, the marking was unlikely to affect the behaviour, fitness or survival of the lizards.

### Morphological measurements and body temperature

A flexible measuring tape was used to determine snout-vent length (SVL) and total length (TL). Head length (HL), head width (HW), head depth (HD), forelimb length (FLL) and hindlimb length (HLL) measurements were made using digital calipers. Body weight was measured with a spring scale (Pesola^®^). An EBRO^®^ Compact J/K/T/E Thermocouple Thermometer was used to obtain all temperature readings (model EW-91 219–40; Cole-Parmer, Vernon Hills, IL, USA). Core body temperatures were recorded from the cloaca using the probe T PVC epoxy tip 24GA, usually within five minutes of capture. Finally, sex was determined on the basis of external sexual dimorphism: males are larger and have two white bands on each side of the body, while females are smaller with orange coloration on the ventral–frontal area (Troya-Zuleta, 2012).

### Blood sample collection and handling

Within five to ten minutes of capture, each lizard was manually restrained while 0.03–0.2 ml of blood was obtained from the coccygeal vein or the heart using a heparinized 31–29 gauge needle attached to a 1.0-ml syringe. The blood was immediately divided into several samples: (1) for making blood films on clean glass microscope slides, (2) for filling microhaematocrit tubes and (3) for loading the lactate strip or the CG8+ iSTAT cartridge within 5 min of sample collection. Any remaining blood was stored on ice and then maintained at −20°C in a freezer at the Galápagos Science Center for future analysis. In some cases, we were only able to extract enough blood to prepare slides and fill microhaematocrit tubes.

### Biochemistry parameters

Blood gas, electrolyte and biochemistry results were obtained using an iSTAT Portable Clinical Analyzer (Heska Corporation, Fort Collins, CO, USA) with CG8+ cartridges. The iSTAT is a portable, hand-held, battery-operated electronic device that measures a wide variety of blood gas, chemistry and haematology parameters using only a few drops (0.095 ml) of whole, non-coagulated blood. The following parameters were measured: anion gap, TCO_2_, haematocrit, haemoglobin, sodium (Na), chloride (Cl), potassium (K), ionized calcium (iCa) and glucose. Mean corpuscular haemoglobin concentration (MCHC) was also calculated [Hb (g/L)/Htc (%) * 100].

### Haematology

Heparinized whole blood was stored on ice immediately after collection. Time-sensitive analyses were completed on the day of sampling. Haematocrit was determined using high-speed centrifugation of blood-filled microhaematocrit tubes. Differential white blood cell counts were made by examining 100 white blood cells on a peripheral smear stained with Diff-Quick stain (Campbell, 2014).

### Statistical analysis

We calculated standard summary statistics of all parameters for each sex before testing for differences in morphometrics, biochemistry and haematology between the sexes using *t-*tests. All statistical analyses were run using IBM SPSS.^®^v23 with a standard α level of 0.05.

## Results

### Morphometrics and body temperature

Males were larger than females in all morphometric measurements. There were significant differences between sexes in SVL (*P* < 0.0001), TL (*P* < 0.0001), HL (*P* < 0.0001), HW (*P* < 0.0001), HD (*P* < 0.0001), FLL (*P* < 0.0001) and HLL (*P* < 0.0001) (see Table 1). The mean body mass for males was 23 g (14.5–31 g) and 10 g (6.5–14 g) for females. This difference was also significant (*P* < 0.0001).

**Table 1:** Morphometric measurement of San Cristóbal Lava Lizards (*Microlophus bivittatus*) separated by sex

Morphometrics	Male	Female
	*n* = 25	*n* = 22
TL (mm)	195.7	156.4
	(30.3)	(19.8)
	127.0–237.0	96.0–182
SVL (mm)	80.6	64.7
	(5.3)	(3.5)
	69.6–90.0	57.0–71.8
HL (mm)	16.0	13.8
	(0.9)	(0.9)
	13.7–17.4	11.6–16.0
HW (mm)	12.6	10.2
	(0.96)	(0.9)
	11.1–14.7	7.6–11.8
HD (mm)	10.1	8.2
	0.8	0.8
	8.4–11.8	7.1–10.5
FLL (mm)	33.2	26.2
	2.4	1.7
	29.0–39.0	24.0–29.0
HLL (mm)	53.4	41.8
	3.9	2.5
	46.0–60.0	37.0–47.0

Values represent means, standard deviation (parentheses) and range (minimum–maximum). TL (tail), SVL (snout-vent length), HL (head length), HW (head width), HD (head diameter), FLL (front leg length), HLL (hind leg length).

The mean internal body temperature for males (*n* = 20) was 34.37°C (range 32.4°C–36.1°C). For females (*n* = 17), it was 33.14°C (range 29.3°C–36.2°C).

### Biochemistry parameters

Summary statistics of the biochemistry data are provided in Table 2. The Cl values of 20 individuals exceeded the maximum detectable value of the iSTAT (140 mmol/l). While calculating means for Cl, we used 140 mmol/l for the samples that exceeded the upper limit of the reportable range. Thus, our procedure likely underestimates the mean Cl level for both males and females in the population.

**Table 2:** Blood biochemical values of San Cristobal Lava Lizards (*Microlophus bivittatus*) separated by sex

Analyte	Male	Female
Na (mmolol/l)	*n* = 23	*n* = 9
	165.77	168.26
	(6.99)	(3.66)
	149–180	160–171
K (mmol/l)	*n* = 22	*n* = 9
	4.26	3.51
	(1.90)	(0.97)
	2–8.1	2–4.9
Cl (mmol/l)	*n* = 21	*n* = 9
	138.44	138.52
	(2.87)	(3.12)
	131–140	132–140
iCa (mmol/l)	*n* = 23	*n* = 9
	1.56	1.67
	(0.14)	(0.25)
	1.31–1.9	1.4–2.24
tCO2 (mmol/l)	*n* = 23	*n* = 9
	9.17	10.44
	(2.55)	(2.50)
	6–15	7–14
Glucose (mg/dl)	*n* = 23	*n*= 9
	272.30	241.22
	(45.96)	(19.79)
	199–401	218–273
Lactate (mmol/l)	*n* = 24	*n* = 22
	17.08	17.04
	(2.06)	(2.62)
	13.6–22.2	8.8–21.1
Total protein (mg/dl)	*n* = 22	*n* = 21
	8.10	8.77
	(1.06)	(2.07)
	5–10.2	5–12
Haemoglobin (g/l)	*n* = 22	*n* = 9
	12.14	10.26
	(1.65)	(1.33)
	8.5–14.3	7.8–12.2
Haematocrit (%)	*n* = 24	*n* = 21
	33.39	27.80
	(5.90)	(7.85)
	23–43	8–39
MCHC (g/l)	*n* = 21	*n* = 9
	(37.82)	(44.22)
	7.41	15.2
	27.94–54.78	25.67–75

Values represent *n*, means, standard deviation (parenthesis) and range (minimum–maximum). Sample sizes vary because some females were too small to extract the amount of blood required for all analyses.

**Figure 2:**
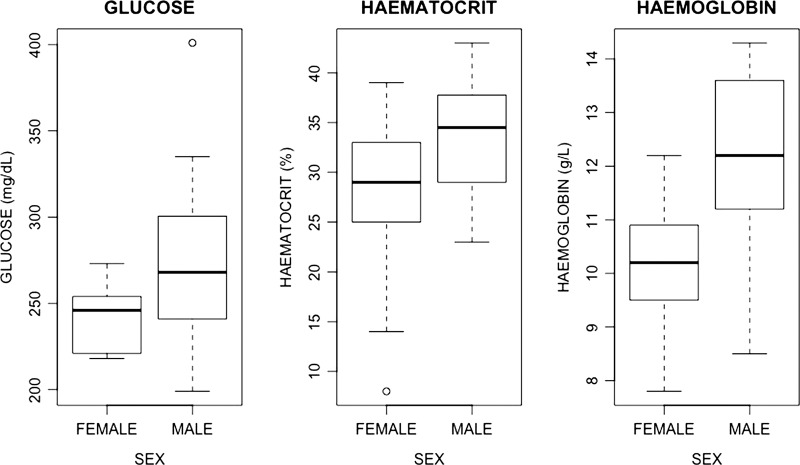
Haematocrit, haemoglobin and glucose box plots for males and females of the San Cristobal lava lizard (*M. bivittatus*). Significant differences were found between sexes. Haematocrit (*P* = 0.009), haemoglobin (*P* = 0.005), glucose (*P* = 0.011).

We looked for sex differences between all the biochemistry analytes but did not find differences in lactate (*P* = 0.95), total protein (*P* = 0.2), Na (*P* = 0.32), K (*P* = 0.27), Cl (*P* = 0.94), iCa (*P* = 0.28), tCO_2_ (*P* = 0.21) and MCHC values (*P* = 0.26). Significant differences were found in glucose values (*P* = 0.011), in haematocrit values (*P* = 0.009) and in haemoglobin (*P* = 0.005). In all three of these cases, values in males were higher than those of females (Fig. 2).

### Haematology

**Figure 3:**
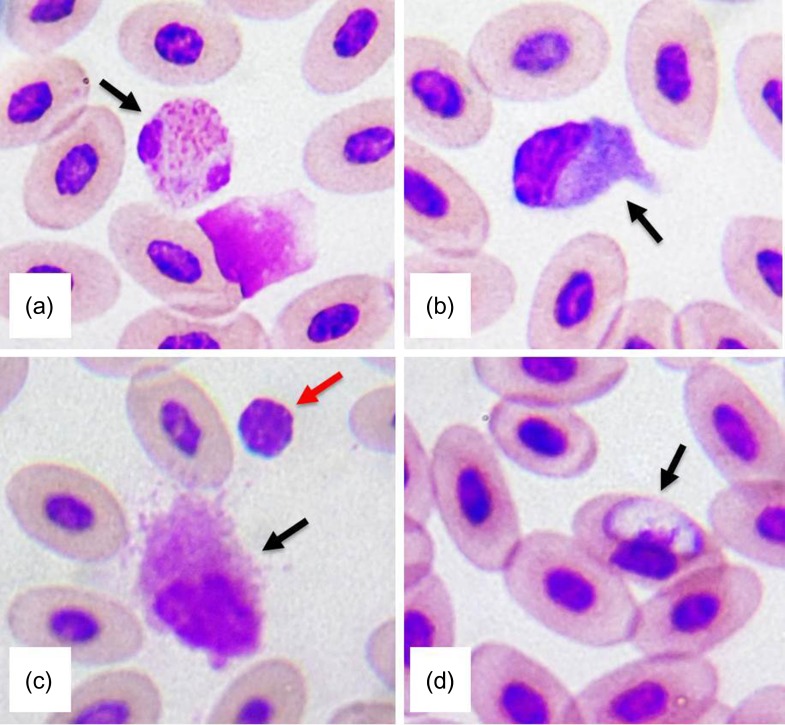
Photographs of selected San Cristóbal lava lizard (*Microlophus bivittatus*) blood cells stained with Diff-Quick stain at 100×. (**a**) Heterophil. (**b**) Monocyte. (**c**) The black arrow indicates an eosinophil and the red arrow indicates a lymphocyte. (**d**) Intraerythrocytic haemoparasite.

Leucocyte values are presented in Table 3. No sex differences were detected for lymphocytes (*P* = 0.49), heterophils (*P* = 0.57) or monocytes (*P* = 0.79). We also noted the presence of an intraerythrocytic haemoparasite while examining the blood slides of one female (Fig. 3).

**Table 3:** Leucocyte counts of San Cristobal Lava Lizards (*Microlophus bivittatus*) separated by sex

Cell type	Male	Female
	*n* = 25	*n* = 22
Lymphocyte (%)	86.04	84.81
	(5.43)	(6.61)
	70–93	70–92
Monocyte (%)	7.16	7.45
	(3.14)	(4.54)
	3–16	0–20
Heterophil (%)	5.4	6.18
	(4.98)	(4.42)
	1–22	0–14
Eosinophil (%)	1.08	0.32
	(1.58)	(0.65)
	0–6	0–2
Basophil (%)	0.64	0.54
	(1.22)	(1.22)
	0–5	0–5

Values represent means, standard deviation (parenthesis) and range (minimum–maximum).

### 

#### Brief descriptions of leucocytes

*Lymphocytes*: Round cells with a round basophilic nucleus, a variable nucleus to cytoplasm ratio and usually non-visible cytoplasm (Fig. 3).

*Monocytes*: Large round to amoeboid cells, nucleus semi-lobed with less condensed chromatin and a pale blue cytoplasm (Fig. 3).

*Heterophils*: Round with eosinophilic granules and a bilobed (sometimes four lobules) nucleus (Fig. 3).

*Eosinophils*: Large amoeboid to round cells with rather purple conspicuous granules and round nuclei (Fig. 3).

*Basophils*: Small round cells with purple granules obscuring the nucleus.

## Discussion

Our results provide the first data on the morphometrics, body temperature, biochemistry and haematology of San Cristóbal lava lizards (*M. bivittatus*). Such baseline data on the morphology and physiology of wild animals is crucial to understanding various aspects of a population’s biology and health (Innis, 2014) and can be especially important in evaluating the cause of morbidity events and population declines.

Morphometrics can be helpful in evaluating cases of disease or environmental stress, as optimal body condition can be calculated using body weight and size (Donoghue, 2006; Lazić *et al.*, 2013). Similarly, the typical ranges of body temperatures and heart rates of a species are critical metrics useful to veterinary professionals during medical interventions (e.g. anaesthesia and surgery). These parameters are known to vary with handling stress in reptiles (e.g. Murray, 2006), so the values reported here should be used only as approximations.

The measurement of biochemical and haematological parameters can serve as a valuable tool for evaluating and monitoring the health of wild reptile populations (Stacy *et al.*, 2011; Campbell, 2014). To determine the significance of changes in biochemical and haematological values associated with factors such as disease, injury, pollutants or starvation, it is essential to establish species-specific (or at least taxon-specific) normal values for parameters of interest (Lewbart *et al.*, 2015). The sole published haematological study of a neotropical ground lizard (*Tropidurus torquatus*) reported only erythrocyte values and morphology (Scorza, 1971), and no data have previously been reported for *Microlophus*.

In general, the biochemistry values reported in Table 2 were very similar to those of other lizards (Divers *et al.*, 1996; Wagner and Wetzel, 1999; Dennis *et al.*, 2001; Harr *et al.*, 2001; James *et al.*, 2006; Maria *et al.*, 2007; Dallwig *et al.*, 2011; Lewbart *et al.* 2015). Because the iStat cannot measure chloride concentrations greater than 140 mmol/l and 20 individuals exceeded this limit, we suspect that chloride levels are high in clinically healthy individuals. We note that similarly high values exceeding 140 mmol/l have been reported in other lizards such as *Pogona vitticeps and Iguana iguan*a (Ellman, 1997; Harr *et al.*, 2001).

For lactate values, we found higher levels than reported by González-Morales *et al.* (2015) in *Sceloporus torquatus*, González-Morales *et al.* (2017) in *S. grammicus*, and Lewbart *et al.* (2015) for marine iguanas (*Amblyrhynchus cristatus)*. Very few studies have reported lactate levels in squamate reptiles (Lewbart *et al.*, 2015; González-Morales *et al.*, 2015, 2017), and most evaluated lactate production after exercise in diving reptiles (Bartholomew *et al.*, 1976; Seymour, 1979; Harms *et al.*, 2003; Warren and Jackson, 2008). In squamates, blood lactate levels may be affected by altitude (González-Morales *et al.*, 2015, 2017), exercise (Bennett *et al.*, 1981; Gleeson and Dalessio, 1989; Schuett and Grober, 2000) and handling (Lewbart *et al.*, 2015). Lactate levels may also be influenced by glucose and oxygen levels (Gleeson and Dalessio, 1989; Bennett, 1973), but apparently there is little dependence on body temperature (Bennett and Litch, 1972). In mammal medicine, hyperlactatemia usually correlates with disease severity and mortality (Gillespie *et al.*, 2016) and with capture myopathy and muscle damage in birds (Burgdorf-Moisuk *et al.*, 2012). Little is known, however, about its clinical use in reptile medicine and further study is needed.

Glucose levels can vary in reptiles depending on nutritional or environmental conditions (Campbell, 2014). In *M. bivittatus* glucose was significantly lower in females than males. Such sex-based variation was not identified in a study of the agamid genus Calotes (Chandavar and Naik, 2012), and the prevalence of this pattern in other lizard families, including Tropiduridae, is unknown. Because glucose metabolism in reptiles is similar to that in mammals, a known difference between human sexes may provide insight (Anish *et al.*, 2013; Mauvais-Jarvis, 2017). Compared to men of the same age, healthy women have lower skeletal muscle mass and higher adipose tissue mass, more circulating free fatty acids, and higher intramyocellular lipid content, all of which might promote insulin resistance in women relative to men (Mauvais-Jarvis, 2017). In addition, insulin sensitivity is greater in women as a result of higher glucose disposal by skeletal muscles (Mauvais-Jarvis, 2017). The mechanisms for facilitated glucose homoeostasis are unclear, but could be due at least in part to the effect of circulating oestrogen (Mauvais-Jarvis, 2017). Although a similar process may explain our findings in *Microlophus*, it is important to note that Dickinson *et al.* (2002) found that male tortoises had higher glucose levels than females, raising questions about whether such patterns are conserved across amniotes.

Blood cell counts and morphology can vary greatly among species of reptile, even among members of the same genus (Stacy *et al.*, 2011; Innis, 2014). In addition, numerous factors, including age, sex, environment, season, presence of environmental stressors, parasite load, nutritional state and restraint, can complicate the evaluation of haematological data in reptiles (Campbell, 2014). Therefore, published reference intervals provide only a baseline for interpretation, and veterinarians need to be aware of these factors to accurately interpret and correlate haematological and clinical findings in reptile patients (Stacy *et al.*, 2011).

Cellular morphology of *M. bivittatus* is similar to that reported in the closely related families Iguanidae, Corytophanidae, and Liolaemidae. As in other lizards that have been studied [e.g. *Cyclura nubila*, (Alberts *et al.*, 1998); *Amblyrhynchus cristatus* (Lewbart *et al.*, 2015); *Salvator* (*Tupinambis*) *merianae* (Troiano *et al.*, 2008); *Pogona vitticeps* (Ellman, 1997)], the highest leucocyte population was the lymphocytes.

We could not analyse total erythrocyte counts due to logistical constraints. However, haematocrit, haemogoblin and MCHC were run as references for red cell values. Lizard haematocrits tend to be higher than those of other reptiles (Campbell, 2014), and lava lizard values resembled those reported in other lizards. Differences were found between sexes; males had higher values, a phenomenon previously reported in reptiles (Harr *et al.*, 2001). It is important to know normal values and differences between sexes to identify possible disease events. For example, low haematocrit may be due to anaemic processes (haemorrhagic, haemolytic or decreased red cell production), but a higher than normal value may be due to haemoconcentration, usually as a result of dehydration. Haemoconcentration also sometimes involves altitude (He *et al.*, 2013; González-Morales, 2015, 2017) or body size and age (Dunlap, 2006). Anaemia has been reported in free-living birds and mammals, but is also often associated with traumatic events or ill health (e.g. severe dehydration or nutritional stress, toxicity, or high blood loss due to parasites (Williams *et al.*, 2004). However, a decrease in haematocrit has also been observed in birds during ‘normal’ reproduction. Specifically, haematocrit has been found to drop routinely between pre-laying and egg-laying, possibly due to plasma vitelogenin levels, estradiol effects on erythropoiesis, nutritional stress and temperature (Williams *et al.*, 2004). Similar processes may drive the differences observed in lizards.

The presence of hemoparasites in wild reptiles is common and is usually considered non-pathogenic (Stacy *et al.*, 2011). The fact that just one individual had hemoparasites (an unknown species of haemogregarine) is surprising. It might be due to limitations of sample size, parasite prevalence, and susceptibility or resistance to infection of particular individuals (Maia *et al.*, 2014). A study in wall lizards in Portugal with a similar sample size reported more than 50% prevalence using microscope diagnostics (Maia *et al.*, 2014), suggesting that *M. bivittatus* might actually have low prevalence. Because hemoparasites can infect multiple species of sympatric reptile (Maia *et al.*, 2014), the relatively low reptile diversity of San Cristobal might limit hemoparasites on the island.

One caveat about our study is that the iSTAT unit used to analyse blood chemistry was designed as a fast and efficient way to determine blood gas and chemistry parameters in humans. For this reason, future work should involve corroborating these results with methods that are more commonly used for reptile work in veterinary laboratories (Steinmetz *et al.*, 2007; Harter *et al.*, 2015), which was impractical in our study due to export constraints and logistical challenges.

In sum, our study represents an initial step toward producing a foundation for future health assessment work on *Microlophus* lava lizards, a small and interesting group of reptiles in the Galápagos. Because there are several closely related species on various islands in the Archipelago, future studies should determine the extent to which the parameters measured here vary across species.
